# Marginal Orbicularis Oris Hyperactivity (MOOH): An Exploratory Case Series of Inversion-Dominant Upper Lip Dynamics Following Intraoral Botulinum Toxin Type A

**DOI:** 10.3390/toxins18030146

**Published:** 2026-03-17

**Authors:** Andrea Felice Armenti

**Affiliations:** LA VISION Training Institute, Via Magenta, 5, 00185 Rome, Italy; andrea.armenti@lavisiontin.com

**Keywords:** botulinum toxin type A, orbicularis oris muscle, upper lip dynamics, vermilion inversion, inversion-dominant phenotype, lip abnormalities, gingival display, intraoral injection

## Abstract

Excessive gingival display (EGD) is most commonly attributed to vertical upper lip elevation, short lip length, or dentoalveolar disproportion. However, some patients exhibit gingival or mucosal visibility during smiling despite minimal vertical upper lip displacement, suggesting the presence of alternative dynamic patterns. This exploratory case series examined an inversion-dominant smile presentation and its documented modulation in association with conservative intraoral chemodenervation directed toward the marginal region of the orbicularis oris. Ninety-four patients with dynamic inversion during smiling were assessed using a qualitative rating aid (Armenti Inversion Scale, AIS) and a quantitative composite proportional measure of vermilion loss (ΔLv%). All patients received low-dose intraoral Onabotulinum toxin A (4–6 U) as part of routine aesthetic care. At Day 15 follow-up, a shift toward lower inversion grades was observed across the cohort, with a large proportion of patients showing minimal residual inversion. Quantitative ΔLv% values showed proportional changes that were directionally consistent with shifts in AIS grade. The functional alterations were mild, transient, and self-resolving. Preliminary inter-rater agreement for AIS and measurement repeatability for ΔLv% suggested acceptable internal consistency for exploratory reporting tools. These findings suggest that inward vermilion inversion may represent a potentially distinct dynamic contributor to gingival or mucosal exposure in selected individuals, and that conservative marginal chemodenervation was associated with transient modulation of this pattern with generally preserved oral competence. AIS and ΔLv% are preliminary documentation tools. Further studies incorporating objective neuromuscular assessment, three-dimensional imaging, and comparative designs are required to refine phenotypic characterization.

## 1. Introduction

EGD, commonly known as “gummy smile”, is a common aesthetic concern typically defined as the visibility of more than 2 mm of maxillary gingiva during smiling [1,2,3]. Its etiology is multifactorial and may involve skeletal disproportion, dentoalveolar variations, and soft-tissue or muscular factors, including short upper lip length or hyperactivity of upper lip elevator muscles [1,2,3]. Increasing aesthetic demand and higher prevalence among female patients have contributed to the growing interest in minimally invasive treatment approaches [2,3,4]. A wide range of therapeutic options have been proposed, including esthetic crown lengthening, lip repositioning, orthognathic surgery, and orthodontic intrusion. Among minimally invasive strategies, hyaluronic acid augmentation, fat grafting, and botulinum toxin type A (BoNT-A) chemodenervation are commonly employed [2,3,4]. Systematic reviews have shown that BoNT-A injections targeting the upper lip elevator complexes can transiently reduce gingival exposure by limiting vertical lip elevation, with a generally favorable safety profile when conservative dosing protocols are applied [5,6,7,8].

Beyond elevator modulation, BoNT-A has also been used to influence orbicularis oris for aesthetic purposes such as elongation of the vermilion, contouring of the lips, and perioral balance [9,10,11]. More recent classifications of gummy smiles have acknowledged that inward lip inversion may accentuate gingival or mucosal visualization in selected cases [9,12]. Nevertheless, inversion has generally been framed as a secondary or accessory component within elevator-dominant presentations, and remains poorly characterized as a primary dynamic pattern. Currently, there are no validated diagnostic criteria or standardized workflows to characterize inversion-dominant smile mechanics or to stratify patients for selective marginal modulation. Based on these observations, the present exploratory study investigates an inversion-driven presentation, tentatively termed marginal orbicularis oris hyperactivity (MOOH), characterized by centripetal rolling of the upper vermilion during smiling in the presence of minimal vertical displacement, as a descriptive construct. In the absence of electromyographic assessment, the term ‘hyperactivity’ is used here as a descriptive clinical construct to indicate visually apparent inversion-dominant overactivity of the marginal orbicularis oris during smiling, rather than as a direct physiological measurement of muscle tone.

A combined qualitative and quantitative documentation workflow—comprising the AIS and the ΔLv%—was applied to descriptively document inversion-dominant upper lip dynamics. AIS and ΔLv% are introduced as preliminary documentation tools to structure clinical reporting of inversion-dominant dynamics.

## 2. Results

### 2.1. Study Population and Baseline Characteristics

A total of 94 consecutive patients met the inclusion criteria for inversion-dominant presentation and were included in the analysis. The baseline demographic characteristics, the AIS distribution, and the quantitative inversion values of the vermilion are summarized in Table 1. At baseline, 53/94 patients (56.4%) presented severe inversion (AIS 3), 24/94 (25.5%) had moderate inversion (AIS 2), and 17/94 (18.1%) mild inversion (AIS 1). All patients exhibited gingival or mucosal exposure during smiling, which was the presenting feature that prompted clinical evaluation rather than a uniform therapeutic target. Variable degrees of vertical upper lip excursion were observed between the cohort: 54/94 patients (57.4%) demonstrated vertical displacement ≤ 2 mm, while 40/94 patients (42.6%) exhibited displacement > 2 mm. The vertical excursion of the upper lip was recorded for descriptive purposes only and was not targeted by the intervention (Table 2). All 94 included patients completed the Day 15 assessment.

### 2.2. Primary Outcomes

#### 2.2.1. Qualitative Inversion Trend (AIS)

On Day 15 after initial conservative dosing, no patients were classified as AIS 3. Residual AIS 2 was observed in 11/94 patients (11.7%), while 54/94 (57.4%) shifted to AIS 1 and 29/94 (30.9%) achieved minimal inversion (AIS 0). These grade distributions are reported in Table 3.

At 6-months of follow-up, 72/94 patients (76.6%) maintained AIS 0 or AIS 1. Twenty-two patients received maintenance dosing following the same predefined escalation parameters. At 12 months, 68/94 patients (72.3%) remained at AIS 0 or AIS 1, while 26/94 (27.7%) received additional maintenance dosing. Follow-up assessments at 6 and 12 months were not designed to evaluate the duration of BoNT-A effect or treatment efficacy, as these timepoints were influenced by maintenance retreatment; these were included exclusively to document functional safety, reversibility, and absence of delayed adverse events.

#### 2.2.2. Quantitative Vermilion Trend (ΔLv%)

ΔLv% values reported in this section represent composite regional measurements derived from the central and bilateral lateral vermilion segments, as defined in the Methods. The baseline ΔLv% averaged 80 ± 12% (median 81%, IQR 72–86%). The quantitative results at all time points are presented in Table 4. On Day 15, the mean ΔLv% decreased to 40 ± 10% (median 39%, IQR 33–47%), corresponding to a proportional reduction in the composite ΔLv% value. At 6 months, the mean ΔLv% was 45 ± 11.4% (median 44%, IQR 36–53%), and at 12 months, it measured 50 ± 12.8% (median 49%, IQR 40–58%). This reduction reflects a proportional decrease in visible vermilion inversion rather than an absolute linear change.

Values at 6 and 12 months reflect maintenance-supported follow-up and should not be interpreted as pharmacological duration. Stratification by baseline AIS demonstrated proportional response magnitudes (Table 5). Patients with baseline AIS 3 exhibited a reduction in mean ΔLv% from 84% to 45%, AIS 2 cases from 76% to 36%, and AIS 1 cases from 64% to 18%. These changes characterize the direction and relative magnitude of modulation.

#### 2.2.3. Dose-Related Modulation Patterns

All patients received an initial total dose of 4 U. Escalation to a total dose of 6 U occurred in 11/53 patients (20.8%) with baseline AIS 3 under predefined conservative criteria. Among patients with baseline AIS 2, 12/24 (50.0%) were descriptively classified as AIS 0 on Day 15 without escalation, under a standardized initial dosing approach. Detailed dose-related modulation patterns are summarized in Table 6. Maintenance treatments were administered at 6 months in 22/94 patients (23.4%) and at 12 months in 26/94 patients (27.7%). No escalation was performed beyond a total dose of 6 U at any time. No qualitative differences in the type, frequency, or persistence of functional events were observed between patients maintained on 4 U and those requiring escalation to 6 U.

### 2.3. Secondary Aesthetic Outcomes

#### 2.3.1. Mucosal Exposure

All patients demonstrated gingival or mucosal exposure at baseline (Table 3). On Day 15, disappearance of mucosal exposure was documented in 62/94 patients (66.0%). The absence of mucosal exposure was observed in 58/94 patients (61.7%) at 6 months and in 49/94 patients (52.1%) at 12 months. These values reflect maintenance-supported follow-up.

#### 2.3.2. Symmetry Patterns

Symmetry improvement was observed across baseline inversion grades, without a clear severity-dependent pattern.

#### 2.3.3. Patient-Reported Experience

Study-specific patient-reported outcome (SS-PRO) scores demonstrated an improvement in aesthetic satisfaction and smile confidence without deterioration in functional comfort (Table S1). Mean aesthetic satisfaction increased from 2.3 ± 1.1 at baseline to 7.8 ± 1.4 on Day 15, while self-perceived inversion decreased from 8.7 ± 1.2 to 3.2 ± 0.9. Items related to speech and suction competence remained stable.

The composite aesthetic–functional results are presented in Table S2. In general, 26/94 patients (27.7%) achieved an “optimal” outcome and 48/94 (51.0%) an “effective” outcome. Three patients (3.2%) were classified as having “undesirable” outcomes related to transient dysfunction or limited response. All reported functional symptoms were transient and considered acceptable by patients; no participant reported symptoms as unacceptable, requested treatment discontinuation, or declined retreatment for this reason.

#### 2.3.4. Reliability and Internal Consistency Analyses

The agreement between the authors on the classification of the AIS demonstrated substantial preliminary reliability in the 40-case subset, with a weighted Cohen’s kappa κ = 0.82 (95% CI: 0.73–0.90). The repeatability of ΔLv% measurements showed high intra-rater agreement, with an intraclass correlation coefficient of ICC = 0.93 (95% CI: 0.88–0.96) based on repeated assessments in 30 patients.

Internal consistency analyses demonstrated a moderate-to-strong monotonic association between baseline AIS grades and baseline ΔLv% values, as well as directionally coherent associations between AIS grade shifts and proportional ΔLv% changes from baseline to Day 15 (Table S3).

#### 2.3.5. Sensitivity Analysis for Alternative Vertical Excursion Thresholds

Descriptive sensitivity analyses using alternative operational thresholds for vertical upper lip excursion (≤1.5 mm and ≤2.5 mm) yielded comparable inversion grading distributions and quantitative ΔLv% trends (Table S4). These exploratory sensitivity analyses did not alter the descriptive interpretation of inversion-dominant dynamics and are reported to support descriptive consistency rather than subgroup differentiation. The distribution of the cases across the thresholds is summarized in Table S4A, while the qualitative and quantitative results at Day 15 are reported in Table S4B. These findings indicate that the observed inversion-dominant modulation patterns remained descriptively consistent across reasonable variations in the operational vertical excursion cutoff.

#### 2.3.6. Functional Safety

Functional monitoring focused on consonant articulation of the bilabia, liquid retention, oral competence, and kissing dynamics. The functional events ranged from mild to moderate and resolved themselves (Table S5). The most frequently observed transient events included altered bilabial articulation (8/94, 8.5%), kissing alteration (5/94, 5.3%), drooling (4/94, 4.3%), and transient difficulty with liquid retention (3/94, 3.2%). The mean duration of the event ranged from 7 to 16 days, and no persistent deficits were observed.

The functional safety stratified by recovery category is presented in Table S6. Patients who demonstrated recovery ≥80% ΔLv% recovery were descriptively associated with fewer adverse functional events compared to those who showed recovery <60%. These observations describe the functional profile of reversible chemodenervation.

### 2.4. Representative Clinical Cases

Two representative cases illustrating inversion-dominant smiling and its transient modulation following conservative intraoral marginal chemodenervation are presented.

**Figure 1:**
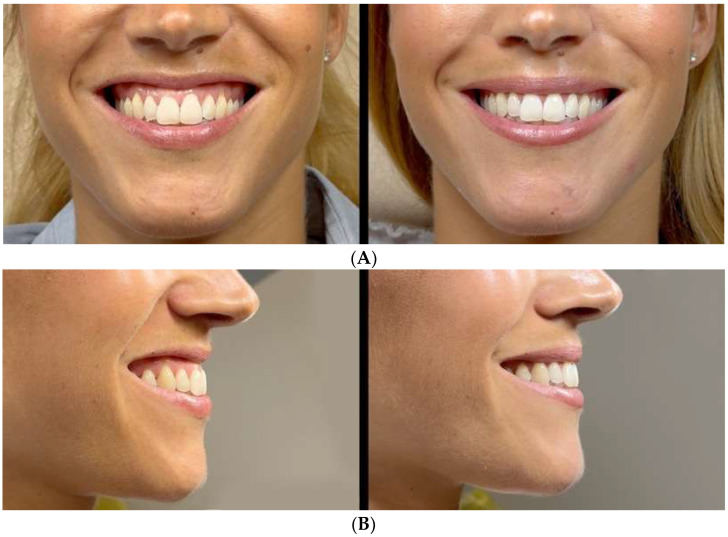
(**A**) Case 1–Frontal View. (**B**) Case 1–Lateral View. 27-year-old female patient with AIS 3; ΔLv% = 82%, documenting transient modulation after conservative marginal intraoral chemodenervation (final AIS 0; ΔLv% = 18%) with preserved oral competence at 6-month follow-up.

Case 1–27-year-old female (Figure 1A,B): The baseline inversion was classified as AIS 3 (ΔLv% = 82%). Following 4 U treatment (four injections of 1 U), minimal inversion was observed on Day 15 (AIS 0; ΔLv% = 18%) with preserved oral competence. Transient modulation persisted at the 6-month follow-up without functional complaints. The corresponding documentation is provided in Video S1a (Case 1—PRE) and Video S1b (Case 1—POST) in Supplementary Materials.

**Figure 2:**
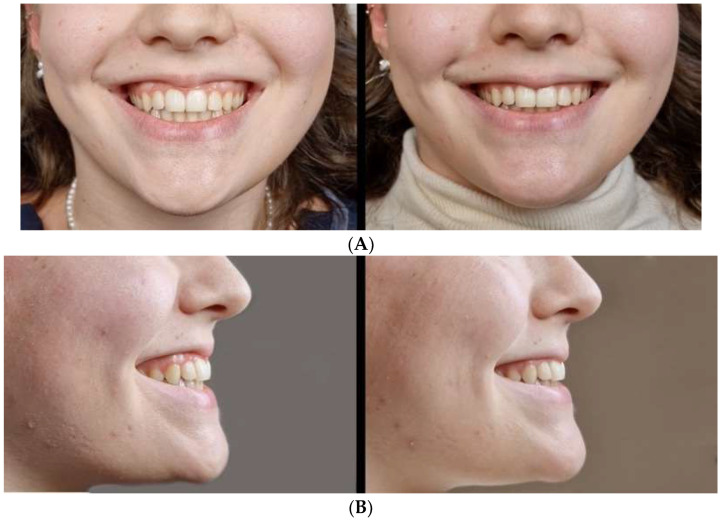
(**A**) Case 2–Frontal View. (**B**) Case 2–Lateral View. 31-year-old female patient with AIS 2; ΔLv% = 78%, partially responsive to initial conservative dosing and documented additional change (final AIS 0; ΔLv% = 22% at 6-month follow-up) following conservative dose adjustment (6 U), as part of the predefined descriptive workflow.

Case 2–31-year-old female (Figure 2A,B): The baseline inversion was AIS 2 (ΔLv% = 78%). Partial modulation was observed on Day 15 after 4 U (AIS 2; ΔLv% = 51%) without impairment of competence. The conservative increase to 6 U resulted in minimal inversion at follow-up (AIS 0; ΔLv% = 22%). A transient self-resolving alteration in kissing was reported and resolved within 14 days. The corresponding documentation is provided in Video S2a (Case 2—PRE) and Video S2b (Case 2—POST) in Supplementary Materials.

Representative dynamic changes in inversion during smiling are illustrated in Videos S1 and S2. These cases illustrate inversion-dominant dynamics and their documentation using AIS and ΔLv%.

## 3. Discussion

EGD has traditionally been interpreted within diagnostic frameworks dominated by skeletal disproportion, dentoalveolar alterations, and hyperactivity of the upper lip elevator muscles [1,2,3]. This conceptual model underlies the widespread use of BoNT-A to reduce gingival exposure by limiting vertical lip elevation, with multiple systematic reviews reporting favorable results on Day 15 when chemodenervation targets the levator complexes [4,5,6]. Implicit in these approaches is the assumption that the visibility of the gingival during smiling primarily reflects the upward excursion of the upper lip.

The present descriptive case series indicates that this assumption may not fully account for all clinical presentations. In a subset of patients, gingival and mucosal exposure was observed despite minimal vertical upper lip displacement, with inward rolling of the upper vermilion representing the dominant dynamic component during smiling. Although recent classifications have acknowledged lip inversion as a contributing factor to EGD [9], such presentations remain poorly characterized and are not operationalized within current diagnostic or therapeutic frameworks. The cohort examined here was intentionally selected to emphasize inversion-dominant dynamics rather than elevator-driven hypermobility or short-lip phenotypes. In this context, MOOH is proposed as a descriptive label to support structured observation of inversion-dominant behavior within existing smile classifications. Taken together, these observations suggest that not all clinically relevant gingival or mucosal exposure during smiling is driven by vertical upper lip excursion, and that inversion-dominant dynamics can be considered a separable functional dimension within the broader spectrum of smile mechanics.

From a mechanistic standpoint, any anatomical or biomechanical interpretation of these observations remains speculative. However, the documented modulation of inversion dynamics is consistent with the existing experimental and biomechanical literature on perioral function. Clinical and modeling studies indicate that localized modulation of the orbicularis oris can influence vermilion contour and lip torque without necessarily inducing substantial cranial displacement of the upper lip [7,8,9,10]. Electromyographic investigations further suggest that perioral muscle activity may be regionally modulated while preserving global functional integrity, depending on dose and diffusion characteristics [11]. In this context, MOOH is best understood as a clinically observed inversion-dominant activity pattern rather than a physiologically validated alteration in muscle tone.

Within this conceptual framework, the present findings are compatible with conservative marginal modulation being associated with changes in inversion dynamics in selected presentations. Preservation of perioral function remains a central concern when modulating the orbicularis oris. Previous electromyographic and clinical investigations indicate that restricted orbicularis modulation can occur without compromising oral competence when toxin diffusion is anatomically constrained [11,12]. Consistent with these observations, the functional alterations in the present cohort were mild, transient, and self-resolving. Longer-term follow-up should therefore be interpreted in terms of safety and reversibility rather than pharmacological duration.

From a clinical perspective, and strictly within the limits of descriptive reasoning, recognizing inversion-dominant behavior as a primary descriptive feature may help clinicians distinguish presentations in which marginal modulation can be explored without directly intervening in elevator muscle activity. This clinical framing must also be interpreted in light of broader functional variables that influence perioral performance.

Age-related changes in perioral performance represent a relevant contextual factor when interpreting smile dynamics and functional safety. In particular, orbicularis oris strength and endurance have been shown to be significantly lower in elderly adults compared with younger adults, suggesting a potential reduction in perioral functional reserve with aging [13,14]. Within this context, the interaction between age-related functional reserve and botulinum toxin exposure warrants careful framing. Large safety syntheses support a generally favorable safety profile of BoNT-A when used conservatively [15,16]. The present cohort consisted exclusively of young adults, and no inference can be drawn regarding elderly populations with potentially reduced neuromuscular reserve. Whether repeated but conservative neuromodulatory interventions may influence longer-term motor patterns remains speculative and warrants dedicated longitudinal investigation [17,18].

The combined use of AIS grading and composite ΔLv% quantification provided a coherent framework for documenting inversion-dominant dynamics. AIS serves as a structured visual grading aid, while ΔLv% offers a proportional metric to standardize quantitative reporting. These tools should be interpreted as documentation instruments rather than validated diagnostic or decision-making criteria. The observed concordance between qualitative grading and quantitative measurement, together with preliminary reliability analyses, is compatible with internal consistency within this exploratory context.

Because ΔLv% is a proportional metric, its clinical meaning lies in the relative redistribution of visible vermilion during smiling rather than in absolute millimetric displacement. A reduction in ΔLv% corresponds to a visible shift from inward rolling toward greater vermilion exposure, even when absolute linear changes cannot be expressed in millimeters. In this sense, ΔLv% complements qualitative AIS grading by enhancing documentation clarity.

In the broader literature on BoNT-A for excessive gingival display, most clinical series have focused on chemodenervation of the upper-lip elevator complex, with heterogeneous injection patterns and short-term assessment windows centered around early follow-up (typically within the first 2–4 weeks) [19,20,21,22]. Reported adverse effects are generally transient and dose-dependent, with variability according to injection site [21,22,23,24].

The absence of a control group, an untreated comparator, or a direct comparison with established elevator-based protocols precludes any assessment of relative effect magnitude or treatment specificity. In particular, the present findings cannot be interpreted as superior, equivalent, or complementary to classic approaches targeting upper lip elevators, which have been widely reported to reduce gingival exposure through a different biomechanical mechanism. Beyond design-related constraints, interpretative boundaries also apply to the measurement tools employed.

Importantly, neither AIS nor ΔLv% should be interpreted as validated diagnostic measures or decision-making criteria. Their value lies in standardizing the observation and enabling reproducible documentation within exploratory investigations. The deliberate inclusion of patients with vertical upper lip excursion greater than 2 mm reflects the phenotypic reality of inversion-dominant presentations and was an intentional methodological choice. As explicitly discussed during the consent process, the therapeutic and analytical focus of the present study was restricted to the dynamics of vermilion inversion, to avoid confounding effects related to elevator muscle treatment, and to preserve interpretability within a clearly defined functional domain.

A further deliberate methodological choice was the adoption of a standardized initial dose irrespective of the severity of the AIS at the start of the study. This approach prioritized functional safety in a competency-critical muscle and minimized dose-related heterogeneity. Dose escalation was limited to predefined conservative adjustment at Day 15 when residual inversion remained clinically relevant. Future comparative studies may explore phenotype-adjusted dosing strategies incorporating objective neuromuscular assessment.

Overall, this work supports the recognition of inversion-dominant smile mechanics as a distinct descriptive dimension and provides a preliminary framework for its structured documentation. The deliberate selection of patients with inversion-dominant dynamics represents a phenotype-focused sampling strategy rather than an attempt to characterize the broader population of patients with mixed or elevator-driven gingival smiles, and therefore introduces an inherent selection bias that limits generalizability. Conservative intraoral marginal chemodenervation was associated with transient modulation of this pattern and largely preserved function.

## 4. Conclusions

This exploratory case series identifies an inversion-dominant smile presentation characterized by centripetal vermilion rolling in the presence of minimal vertical upper lip displacement. In selected individuals, excessive gingival and mucosal display may occur in association with prominent centripetal vermilion inversion, even in the absence of marked vertical upper lip elevation. In such presentations, inward rolling of the upper vermilion appears to represent one dynamic contributor within the broader spectrum of smile mechanics. Within this selected phenotype, conservative marginal chemodenervation was associated with short-term modulation of inversion dynamics while generally preserving oral competence. These findings support the recognition of inversion-dominant smile mechanics as a distinct descriptive dimension within smile analysis and provide a preliminary framework for its structured documentation. Further controlled and comparative studies are required to clarify clinical relevance, underlying mechanisms, and optimal patient selection.

## 5. Limitations

This study has several important limitations that warrant careful consideration. First, the non-comparative single-center design precludes direct comparison with elevator-based or mixed treatment strategies and limits assessment of relative treatment effects. Second, phenotype assignment was based on structured clinical inspection, and both AIS and ΔLv% remain non-validated descriptive tools. Although inter-rater agreement for AIS demonstrated substantial reliability in the evaluated subset, phenotype assignment and grading ultimately relied on structured clinical judgment. The use of observer consensus mitigates, but does not eliminate, the inherent subjectivity associated with visual classification of dynamic facial patterns. Although preliminary reliability analyses were performed, formal validation, external replication, and multicenter assessment are required.

Third, the study design did not allow for the determination of neuromuscular causality. The use of two-dimensional imaging does not capture three-dimensional lip contour changes, and objective neuromuscular assessment was not performed. Dose adjustment and maintenance retreatment may introduce confounding between recovery magnitude and functional observations. In addition, placebo effects, spontaneous variability, and regression to the mean cannot be excluded in a non-comparative design.

Finally, modulation patterns were influenced by maintenance dosing, and no inference can be made regarding long-term structural modification or durable treatment strategies. The relatively narrow age range limits extrapolation to elderly populations with potentially reduced neuromuscular reserve.

## 6. Materials and Methods

### 6.1. Study Design and Ethical Approval

This study was conducted as a single-center observational case series documenting inversion-dominant upper lip behavior in association with conservative intraoral marginal chemodenervation. The study was conducted between January 2023 and March 2024. The protocol adhered to the Declaration of Helsinki and Good Clinical Practice guidelines. Written informed consent was obtained from all participants and included authorization for the anonymized use of images and videos.

The primary descriptive domain of the study was the observed variation in inversion classification assessed using the AIS and the quantitative ΔLv% metric, both used exclusively as descriptive documentation tools rather than validated clinical outcome endpoints. No a priori clinically relevant threshold of change was defined for AIS or ΔLv%, as the aim of these measures was descriptive documentation rather than the quantification of treatment effect; establishing clinically meaningful cutoffs would require external validation and comparative designs beyond the scope of the present study. Secondary descriptive domains included symmetry, mucosal exposure, patient satisfaction, dose increase, and functional safety. The dosing strategy reflected routine clinical decision-making and was not intended as an interventional trial protocol.

### 6.2. Patient Selection and Inclusion Criteria

Patients were eligible for inclusion if they met all the following pre-specified criteria:

- Dynamic inward vermilion inversion during maximal posed Duchenne smile, confirmed by standardized video documentation.
- Visible mucosal exposure associated with inversion.

Vertical upper lip excursion was recorded at rest and during maximal smile using philtral midline calibration but was not used as an exclusion criterion. The vertical elevation of the upper lip, when present, was not targeted by the intervention and was explicitly discussed with patients during the consent process. Exclusion criteria included previous perioral surgery or trauma, neuromuscular disease, active infection, prior BoNT-A treatment within 12 months, pregnancy or breastfeeding, and known toxin hypersensitivity.

The assignment of the clinical phenotype was performed independently by two trained clinicians based on overall inversion-dominant characteristics. In cases of discordance, inclusion required consensus agreement. This approach allowed for the inclusion of mixed presentations while preserving a descriptive focus on inversion-dominant mechanics rather than a comprehensive correction of GDS.

During the study period, 105 consecutive patients presenting with excessive gingival or mucosal display were screened. Eleven patients were excluded after the application of predefined exclusion criteria unrelated to vertical excursion, including prior perioral surgery, recent botulinum toxin treatment, or conditions potentially affecting neuromuscular function. The final study cohort therefore consisted of 94 patients and was included in the analysis. No patients were lost to follow-up during the primary descriptive period. Conservative dose escalation at Day 15 was accepted by all eligible patients following informed consent.

### 6.3. Image Acquisition and Quantitative Measurement (ΔLv%)

Standardized frontal photographs and 60 frames-per-second video recordings were acquired at rest and during maximal smile using a fixed camera distance, neutral head posture, and constant lighting conditions. Image calibration was performed using the philtral vertical reference to ensure consistent vertical orientation across images; this reference was used exclusively for image alignment and not to define the regional vermilion measurement sites.

Visible vermilion length was measured at rest and during maximal smile using a regionalized approach designed to capture central, lateral, and combined inversion patterns. Measurements were obtained at three standardized locations: the midline (Lv_C), corresponding to the Cupid’s bow midpoint, and the left (Lv_L) and right (Lv_R) lateral vermilion segments, defined as the midpoint between the ipsilateral Cupid’s bow peak and the oral commissure. For each region, the proportional inversion of vermilion was calculated separately as ΔLv%_C, ΔLv%_L, and ΔLv%_R according to the following formula:

(1) $$\Delta Lv{\%}_{i}=\left[\begin{array}{c} \frac{\left(Lv_{rest},i-Lv_{smile},i\right)}{}\\ Lv_{rest},i \end{array}\right]\times 100$$

where i denotes the central (C), left lateral (L), or right lateral (R) measurement site.

A composite value ΔLv% was then derived as the arithmetic mean of the three regional values (ΔLv%_C, ΔLv%_L, ΔLv%_R) to provide a single descriptive metric that captures the general inversion of the vermilion across the central and lateral regions. For descriptive purposes, inversion patterns were also categorized as predominantly central (C), lateral (L), or combined (CL), based on the regional distribution of vermilion inversion observed during smiling. Unless otherwise specified, all ΔLv% values reported throughout the manuscript refer to this composite regional metric. ΔLv% was intentionally defined as a normalized proportional measure of relative vermilion inversion between rest and smile, rather than as an absolute linear displacement. As such, it is not directly translatable into millimetric values, and no minimally clinically important difference was predefined.

Two independent examiners recorded values of ΔLv%. Both observers were clinicians with specific training in facial aesthetic assessment and perioral dynamics. They were not involved in the original patient classification or treatment delivery and were blinded to dose, follow-up outcomes, and each other’s ratings. As the aim of the analysis was to provide preliminary information on descriptive reliability rather than formal validation, no further stratification by observer background was performed.

Measurement discrepancies greater than 5% prompted a third assessment, with the median value retained for analysis. A measurement discrepancy exceeding the predefined 5% threshold occurred in 2 out of 30 patients (6.7%) in the repeatability subset, prompting a third blinded reassessment before median value retention. Higher composite ΔLv% corresponded to a greater proportional loss of visible vermilion between the central and lateral regions measured during smiling, reflecting a more pronounced inversion-dominant pattern. The 5% discrepancy threshold was adopted as an operational criterion to trigger third-party reassessment and was not derived from prior validation studies or a formal pilot investigation. This cutoff was selected pragmatically to balance sensitivity to meaningful measurement disagreement with feasibility in a descriptive observational setting. The exploratory dataset used for the ΔLv% descriptive analyses is available as Data File: MOOH DeltaLV Exploratory.xlsx in the Supplementary Materials.

### 6.4. Injection Technique and Predefined Dosing Framework

**Figure 3:**
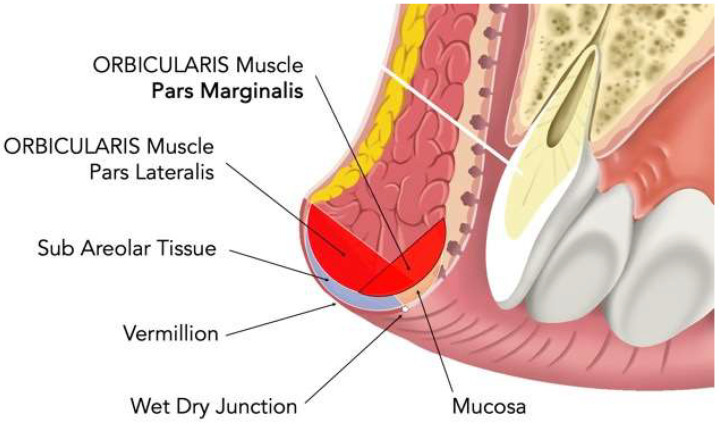
Anatomical schematic of the upper lip highlighting the pars marginalis of the orbicularis oris. Schematic anatomical representation of the upper lip. The image illustrates the pars marginalis and pars lateralis of the orbicularis oris muscle, the subareolar tissue, the vermilion, the labial mucosa and the wet–dry junction. The pars marginalis, highlighted in red, represents the anatomical region explored for conservative marginal chemodenervation in the present descriptive workflow applied to MOOH.

**Figure 4:**
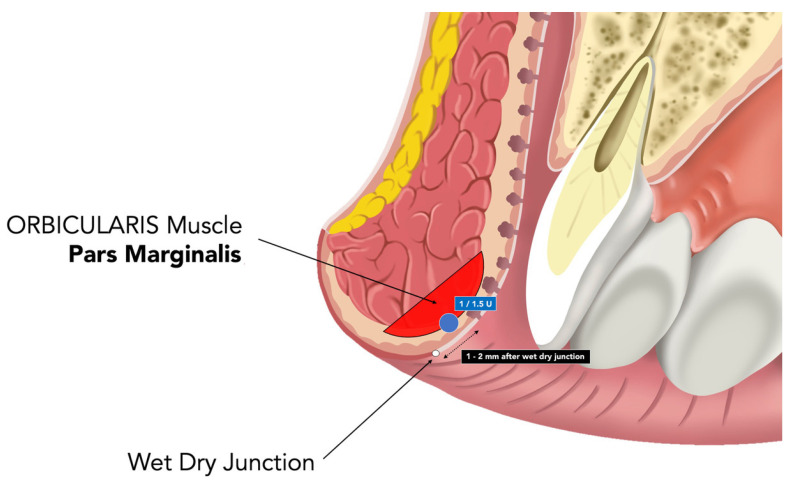
Intraoral orientation for marginal chemodenervation. Schematic representation of the intraoral orientation used to document marginal chemodenervation in MOOH. The illustration shows an intraoral approach through the labial mucosa, approximately 1 to 2 mm below the wet–dry junction, directed toward the marginal region of the orbicularis oris muscle. In the present descriptive workflow, each site received 1 U of Onabotulinum Toxin A as an initial dose (increased to 1.5 U only if necessary at retouch), using a 30—32 G short-bevel needle (≈4 mm) inserted tangentially to a depth of approximately 1.5—2.0 mm. Escalation corresponded to +0.5 U per injection point (6 U total). This schematic is intended solely to illustrate the anatomical orientation of the marginal region explored and does not constitute a procedural guide, recommended technique, or validated injection protocol.

**Figure 5:**
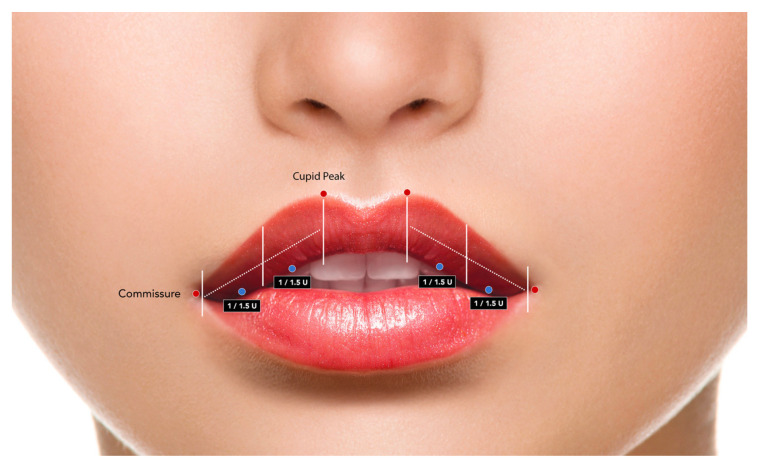
External segmentation and approximate marginal injection sites. External reference scheme illustrating the surface segmentation of the upper lip used for descriptive alignment with regional vermilion assessment in MOOH. The upper lip is divided into two hemi-lips by the midline. On each side, the explored segment extends from the oral commissure to the vertical projection of the ipsilateral Cupid’s bow peak. Each segment is further divided into the medial and lateral halves, corresponding to the regional distribution considered in the descriptive workflow. Red dots indicate anatomical reference points used for orientation (oral commissures and Cupid’s peaks along the vermilion border. Blue dots indicate the approximate location of the marginal intraoral sites explored in this study. Each site received 1 U of BoNT-A as an initial dose, with an optional conservative increase to 1.5 U if required. External segmentation is provided for descriptive alignment with regional vermilion assessment and does not imply a mandatory mapping, algorithm, or dose-distribution rule.

Chemodenervation was performed intraorally at the wet–dry junction, targeting the pars marginalis of the orbicularis oris as a conservative routine approach. The anatomical region explored for conservative marginal chemodenervation is illustrated schematically in Figure 3. Onabotulinum Toxin A (Botox^®^, Allergan Aesthetic, an AbbVie company, Irvine, CA, USA) was reconstituted to a concentration of 2.5 U per 0.1 mL. Four symmetric micro-deposits (two paramedian and two lateral) were administered using a 30–32 G short-bevel needle at 1–2 mm after the wet-dry junction with an approximate depth of 1.5–2.0 mm (Figure 4). The intraoral orientation of the explored marginal region and the relationship with the wet–dry junction are illustrated in Figure 5.

The regionalized ΔLv% evaluation was designed to include the same lateral vermilion segments targeted by the marginal injection protocol, ensuring anatomical and descriptive coherence between quantitative documentation and treatment delivery. The initial dose was intentionally standardized between baseline AIS grades to minimize functional risk and dose-related heterogeneity in this descriptive exploratory workflow; AIS and ΔLv% were not used as dose-titration instruments.

The dosing algorithm consisted of:

- Initial dose: 4 U total (1 U per injection point).
- Optional conservative escalation on Day 15: +0.5 U per injection point if the inversion remained clinically relevant. Accordingly, Day 15 represented both the primary descriptive assessment point and the earliest clinically appropriate decision point for conservative dose adjustment.
- Maximum total dose: 6 U.

Electromyographic guidance was not used and muscle targeting was based on anatomical landmarks and constrained diffusion assumptions.

### 6.5. Outcome Measures and Follow-Up

Day 15 was predefined as the primary descriptive timepoint for outcome assessment, as it reflects the expected peak pharmacological effect of BoNT-A while minimizing confounding related to maintenance treatment. Follow-up visits were scheduled at baseline and on Day 15, with additional assessments at 6 and 12 months when maintenance treatment was clinically requested. Assessments beyond Day 15 were not intended as efficacy endpoints but were performed to document functional safety, reversibility of modulation, and real-world maintenance patterns. Functional safety was assessed at each follow-up visit, focusing on consonant articulation of the lip (p/b/m), liquid suction and retention, oral competence and kissing dynamics. Adverse events were graded using a modified neuromuscular scale derived from the established clinical and neuromuscular BoNT-A literature [11,12]. Aesthetic and functional satisfaction was evaluated using a five-item study-specific patient-reported outcome scale (SS-PRO).

### 6.6. Reliability Assessment

To provide preliminary information on the robustness and reproducibility of the proposed descriptive tools, the inter-rater reliability of the AIS classification and the repeatability of ΔLv% measurements were assessed in randomly selected subsets of the study cohort. For AIS, a random subset of 40 patients was selected using a computer-generated randomization sequence. Two independent clinicians, not involved in the original classification and blinded to treatment dose and follow-up results, independently assigned AIS grades (0–3) based on standardized rest and maximal smile images. The agreement between the authors was evaluated using weighted Cohen’s kappa with quadratic weights.

For ΔLv%, repeatability was assessed in a subset of 30 patients. The same examiner repeated vermilion length measurements after a minimum interval of 14 days, blinded to the initial values. Intra-rater agreement was quantified using the intraclass correlation coefficient (ICC) derived from a two-way mixed-effects model with absolute agreement. No formal sample size or power calculation was performed for reliability analyses, as these were intended to provide preliminary descriptive information on interobserver agreement rather than formal validation; however, the number of paired observations was considered sufficient to yield stable estimates within commonly accepted interpretive ranges for ICC and kappa statistics. These analyses were intended to assess the reliability of preliminary measurements and do not constitute formal validation of AIS or ΔLv% as diagnostic instruments.

### 6.7. Sensitivity Analysis of the Vertical Excursion Cutoff

To assess the robustness of the descriptive findings, vertical upper lip excursion was explored using alternative reference thresholds of 1.5 mm and 2.5 mm. Vertical excursion was recorded as a concomitant descriptive variable and was not used as an inclusion criterion or stratification factor.

Sensitivity analyses were conducted solely to verify whether the observed inversion-dominant patterns and Day 15 modulation trends remained consistent across reasonable variations in the literature-reported reference values for vertical excursion. These analyses were not intended to define diagnostic cutoffs, establish phenotypic subgroups, or support causal inference.

### 6.8. Statistical Analysis

Given the exploratory nature of this study and the investigation of a novel anatomical presentation, the analyses were intended to generate hypotheses rather than estimate effect size or clinical performance. Continuous variables are reported as mean ± standard deviation and median (interquartile range), while categorical variables are expressed as frequencies and percentages. Inferential tests were not performed for clinical efficacy endpoints and no sample size calculation was conducted. However, descriptive measurement and consistency analyses were performed to characterize the proposed reporting tools, including weighted Cohen’s kappa (with 95% confidence intervals) for the agreement of AIS, ICC (with 95% confidence intervals) for ΔLv% repeatability, and Spearman rank correlations (with 95% confidence intervals) to describe the concordance between AIS and ΔLv%.

Supplementary Material S1 provides the standardized documentation workflow and exploratory quantification of vermilion inversion. Supplementary Material S2 contains supplementary tables reporting extended descriptive outcomes, sensitivity analyses, concordance analyses, and detailed functional safety data.

### 6.9. Ethics Statement

The intervention involved the use of Onabotulinum toxin A within the established safety dose ranges but in an anatomical indication not specifically included in the European product labeling. Such use is considered off-label under current regulatory frameworks. Treatment was performed as part of routine aesthetic clinical practice based on individualized assessment.

Clinical data were collected prospectively for documentation and audit purposes without alteration of standard patient management. According to local regulations for observational reporting of routine clinical care, this case series did not require prior ethics committee review; documentation of this determination is available upon request.

All patients provided written informed consent for treatment, including the acknowledgment of off-label use, and for the anonymized use of clinical data, images, and videos for research and publication purposes.

## Figures and Tables

**Table 1:** Demographic and baseline clinical characteristics.

Variable	Total (N = 94)	Females (n = 70)	Males (n = 24)	Notes
Age, years (mean ± SD)	31.0 ± 3.1	30.9 ± 3.0	31.4 ± 3.3	
Age range (years)	27–38	27–38	28–36	Observed range
AIS Baseline Grade 3, *n* (%)	53 (56.4%)	–	–	Severe inversion
AIS Baseline Grade 2, *n* (%)	24 (25.5%)	–	–	Moderate inversion
AIS Baseline Grade 1, *n* (%)	17 (18.1%)	–	–	Mild inversion
AIS Baseline Grade 0, *n* (%)	0 (0%)	–	–	No cases without visible inversion
Baseline ΔLv%, mean ± SD	80 ± 12%	–	–	Quantitative scoring
Mucosal exposure present, *n* (%)	94 (100%)	–	–	All patients showed mucosal exposure
**Inversion pattern, *n* (%)**				
Central (C)	0 (0%)	–	–	–
Lateral (L)	84 (89.4%)	–	–	–
Combined (CL)	10 (10.6%)	–	–	–

Legend: Baseline demographic characteristics and initial inversion severity according to the AIS.

**Table 2:** Distribution of vertical upper lip excursion in the study cohort.

Category	*n* (%)	AIS Categories at Baseline	Role in Analysis
Vertical excursion ≤ 2 mm	54 (57.4%)	AIS 1–3	Descriptive (not treatment-guiding)
Vertical excursion > 2 mm	40 (42.6%)	AIS 1–3	Descriptive (not treatment-guiding)

Legend: Distribution of patients according to vertical upper lip excursion during maximal smile. Vertical excursion was recorded as a descriptive variable and was not used as an inclusion or treatment-guiding criterion.

**Table 3:** Changes in AIS grade over follow-up.

AIS Grade	Baseline (N = 94)	Day 15
3–Severe inversion	56.4%	0%
2–Moderate inversion	25.5%	11.7%
1–Minimal inversion	18.1%	57.4%
0–None	0%	30.9%

Legend: Distribution of AIS grades at baseline and Day 15 following conservative intraoral marginal chemodenervation. Day 15 reflects the standardized initial dosing phase. Six- and twelve-month outcomes are reported descriptively in the text, as they reflect maintenance-supported follow-up rather than uniform assessment intervals. Percentages are shown; absolute counts are available in Supplementary Materials S2.

**Table 4:** Quantitative change in vermilion length (ΔLv%) over time.

Timepoint	ΔLv% Mean ± SD	Median (IQR)	Min–Max
Baseline	80 ± 12%	81% (72–86%)	52–96%
Day 15	40 ± 10%	39% (33–47%)	18–62%
6 Months	45 ± 11%	44% (36–53%)	22–68%
12 Months	50 ± 12%	49% (40–58%)	28–71%

Legend: ΔLv% represents the composite proportional measure of vermilion loss from rest to maximal smile, calculated as the mean of three regional measurements (central and bilateral lateral). Higher values indicate greater inversion. Follow-up values reflect maintenance-supported assessment.

**Table 5:** Quantitative response by baseline AIS.

Baseline AIS	Mean ΔLv% Baseline	Mean ΔLv% Day 15	Proportional ΔLv% Change from Baseline (%)	AIS 0 Conversion n (%)
AIS 3	84%	45%	46%	0 (0%)
AIS 2	76%	36%	52%	12 (50%)
AIS 1	64%	18%	72%	17 (100%)

Legend: ΔLv% change from baseline to Day 15 following conservative intraoral marginal chemodenervation, stratified by baseline AIS. AIS 0 conversion refers to Day 15.

**Table 6:** Dose response by baseline AIS.

Baseline AIS	Patients (n)	Initial Dose 4 U (%)	Escalation to 6 U (%)	AIS 0–Day 15 (%)
AIS 3 (Severe)	53	100%	20.8%	0%
AIS 2 (Moderate)	24	100%	0%	50%
AIS 1 (Mild)	17	100%	0%	100%

Legend: Dose distribution and Day 15 AIS conversion stratified by baseline AIS under a standardized initial dosing protocol. Escalation to 6 U at Day 15 was predefined when residual inversion remained clinically relevant.

## Data Availability

The original contributions presented in this study are included in the article/Supplementary Material. Further inquiries can be directed to the corresponding author.

## Supplementary Materials

The following supporting information can be downloaded at: https://www.mdpi.com/article/10.3390/toxins18030146/s1. Supplementary Material S1: Standardized documentation workflow and exploratory quantification of dynamic vermilion inversion, including photographic and video acquisition parameters, regionalized ΔLv% measurement methodology, illustrative AIS grading examples, and common observational pitfalls. Supplementary Material S2: Supplementary tables reporting extended descriptive outcomes, exploratory concordance analyses, sensitivity analyses of vertical excursion thresholds, and detailed functional safety data. Video S1a: Case 1—PRE; Video S2a: Case 1—POST; Video S2a: Case 2—PRE; Video S1b: Case 2—PRE; Data File: MOOH DeltaLV Exploratory.xlsx.

## Abbreviations

The following abbreviations are used in this manuscript:

MOOH	Marginal Orbicularis Oris Hyperactivity
AIS	Armenti Inversion Scale
ΔLv%	Composite Proportional Measure of Vermilion Loss
