# Supplementary material for: Marginal Orbicularis Oris Hyperactivity (MOOH): An Exploratory Case Series of Inversion-Dominant Upper Lip Dynamics Following Intraoral Botulinum Toxin Type A
Source: Toxins (Basel). 2026 Mar 17;18(3):146. doi: 10.3390/toxins18030146 (PMC13029986; doi:10.3390/toxins18030146)
Supplement: Supplementary file 1 [file toxins-18-00146-s001.zip › Supplementary Material S1.pdf]

Supplementary Material - S1

Structured Documentation and Exploratory Quantification of Dynamic Vermilion Inversion

This appendix describes the non-validated workflow used to document and quantify upper lip vermilion reduction during smiling, in the context of an exploratory investigation of inversion-dominant lip mechanics. The protocol is intended to ensure reproducibility of observation, not to provide diagnostic criteria or treatment indications.

1.Standardized Photographic and Video Acquisition

1.1 Objective

To document upper lip behavior at rest and during maximal Duchenne smiling, with attention to vermillion thinning and inward inversion independently from elevator activity.

1.2 Equipment and Setup

Parameter	Standard
Lens	35–55 mm equivalent
Distance	120–150 cm
Lighting	Diffuse, frontal (avoid shadows affecting vermilion border)
Head Position	Natural Head Position; Frankfort plane horizontal
Background	Neutral, non-reflective
Smile Type	Maximal Duchenne (induced)
Lip Condition	Hydrated, free of makeup, no lip press/pursing

These parameters are provided to enhance reproducibility of observation across cases and centers, not to define mandatory acquisition standards or to exclude alternative documentation setups."

1.3 Standard Smile Cue

"Please smile as if you are genuinely laughing, showing your upper teeth."

1.4 Required Documentation

View	Type	Purpose
Rest	Photo	Baseline vermilion length
Maximal Smile	Photo	Visible thinning/inversion
Smile Dynamics	60 fps 2-second video	Confirmation of inward rolling

Videos serve only to confirm dynamics; they are not measured.

2. Exploratory Quantification of Vermilion Reduction (ΔLv%)

2.1 Definition

ΔLv% represents the proportional reduction in visible upper lip vermilion from rest to maximal smile, assessed using a regionalized approach to account for central, lateral, and combined inversion patterns.

Visible vermilion length is measured at three standardized regions:
– central (Lv_C), corresponding to the midpoint of the Cupid's bow,
– left lateral (Lv_L),
– right lateral (Lv_R),

where lateral measurement points are defined as the midpoint between the ipsilateral Cupid's bow peak and the oral commissure.

For each region, proportional vermilion inversion is calculated separately according to the following formula:

$$\Delta Lv\%_i = \left[\frac{Lv_{rest,i} - Lv_{smile,i}}{Lv_{rest,i}}\right] \times 100$$

where i denotes the central (C), left lateral (L), or right lateral (R) region.

A composite ΔLv% value is then obtained as the arithmetic mean of the three regional values (ΔLv%_C, ΔLv%_L, ΔLv%_R). This composite metric provides a single descriptive measure capturing overall vermilion inversion across central and lateral segments. Pixel values are acceptable because only proportional change is studied.

2.2 Photoshop Workflow (Non-Validated; Reproducibility Target Only)

The following workflow is provided exclusively to illustrate one possible reproducible approach to proportional measurement and is not intended to prescribe a preferred method, software, or measurement standard. The steps below are presented for illustrative purposes only and may be adapted, reordered, or replaced by equivalent procedures without affecting the proportional nature of ΔLv%.

Step A — Side-by-Side Display

- Open both images (rest + smile).
- Arrange side by side:

Window → Arrange → Tile All Vertically

Image S1-A

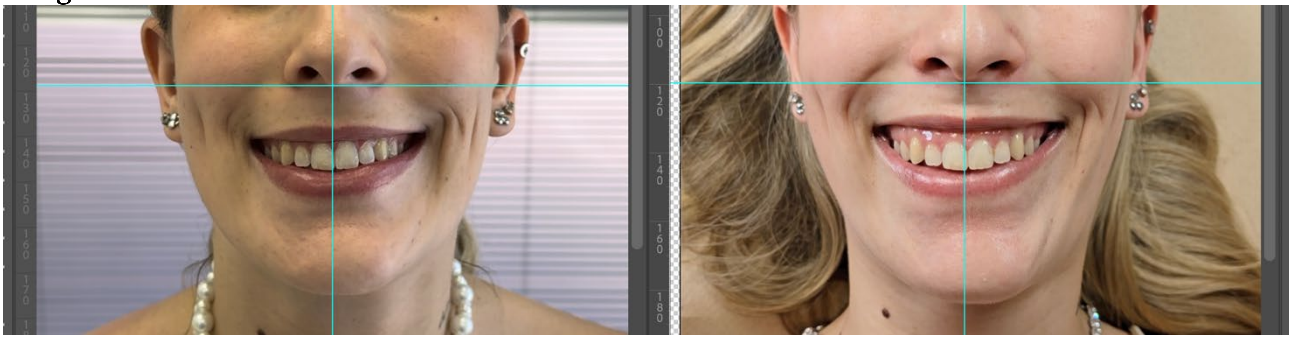

Caption: *Side-by-side documentation prior to measurement. Images are not resized independently.*

Step B — Image Orientation Calibration

- Select the Line Tool.
- Draw a vertical reference line from the base of the columella to the midline of the Cupid's bow.

This reference is used exclusively to standardize vertical orientation across images and does not define the vermilion measurement sites.

Image S1-B

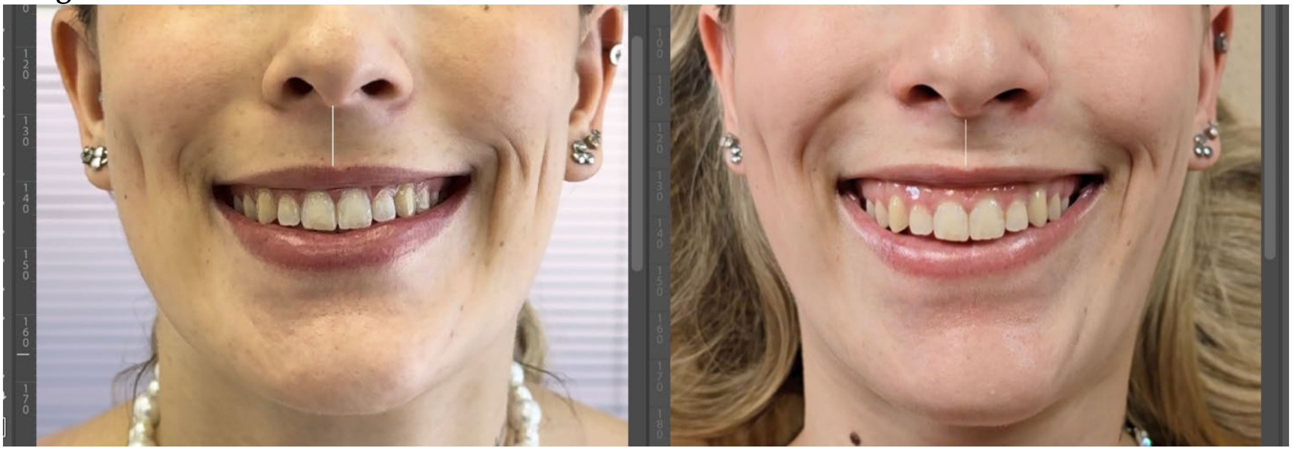

Caption: Midline columella-to-vermilion marker used for vertical calibration reference. No absolute scale is implied.

Step C — Regional Measurement of Visible Vermilion (Red Portion Only)

Measure only the visible red vermilion, excluding:
– exposed mucosa,
– gingival tissue,
– internal wet–dry border extension.

Measurements must be performed at three standardized locations:

- Central region (Lv_C):
- Draw a vertical line from the midpoint of the Cupid's bow to the inferior limit of visible red vermilion.
- Left lateral region (Lv_L):
- Identify the midpoint between the left Cupid's bow peak and the left oral commissure.
- Draw a vertical line from this point to the inferior limit of visible red vermilion.
- Right lateral region (Lv_R):
- Identify the midpoint between the right Cupid's bow peak and the right oral commissure.
- Draw a vertical line from this point to the inferior limit of visible red vermilion.

Hold Shift to ensure vertical alignment for all measurements.

Insert Image S1-D (smile)

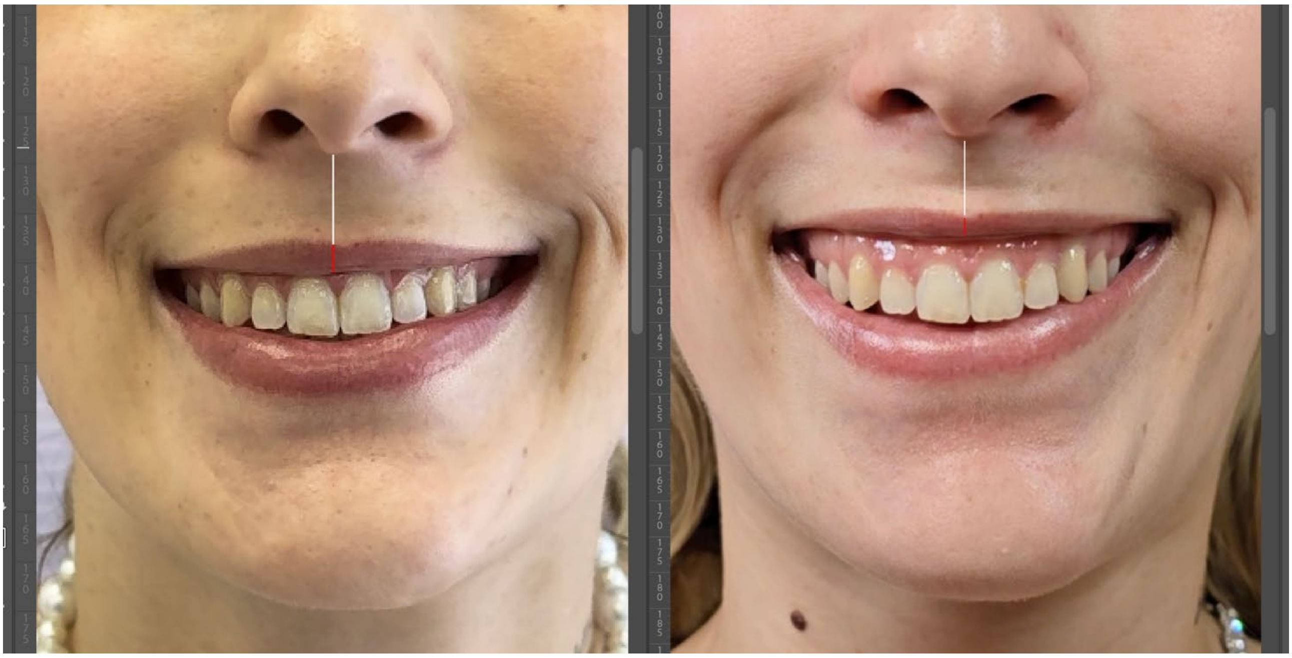

Caption: *Measurement of visible vermilion at maximal smile. Mucosal exposure is excluded.*

Step D — Record Values

- Open: **Window → Measurement Log**
- Click **Record Measurement**.

Record measurements separately for each region (Lv_C, Lv_L, Lv_R) at rest and during maximal smile.

Image S1-E (Measurement Log)

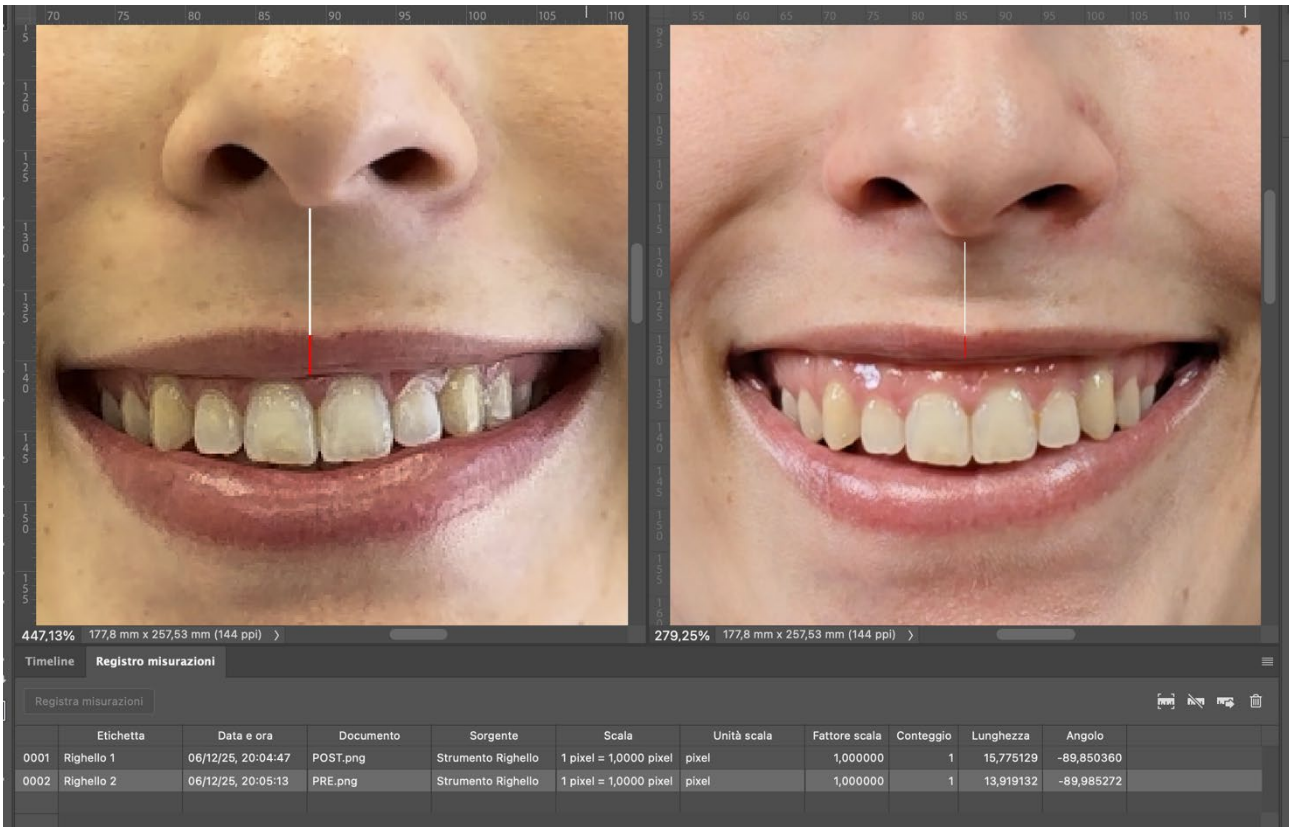

Caption: Illustration of Central Part (Lv_C) only. Pixel values extracted automatically from the Measurement Log. Pixel units are proportional and do not require conversion.

2.3 Example Calculation

Example calculation (regional and composite):

Lv_C_rest = 15.8 px Lv_C_smile = 13.9 px
Lv_L_rest = 14.6 px Lv_L_smile = 11.2 px
Lv_R_rest = 14.9 px Lv_R_smile = 11.5 px

$$\Delta Lv\%_C = \frac{15.8 - 13.9}{15.8} \times 100 = 12.0\%$$

$$\Delta Lv\%_L = \frac{14.6 - 11.2}{14.6} \times 100 = 23.3\%$$

$$\Delta Lv\%_R = \frac{14.9 - 11.5}{14.9} \times 100 = 22.8\%$$

Composite ΔLv% = (12.0 + 23.3 + 22.8) / 3 = 19.4\%

Interpretation:
This patient exhibits a composite ΔLv% of approximately 19%, reflecting a lateral-dominant inversion pattern that would be underestimated by a single midline measurement. This value is descriptive only and does not diagnose a condition or define treatment eligibility.

Software Clarification (Non-Exclusive Procedure)
ΔLv% does not require Adobe Photoshop. Any pixel-based tool (ImageJ, Fiji, GIMP, ruler plug-ins, or smartphone apps) can be used, because ΔLv% expresses proportional change and therefore does not depend on absolute calibration. Photoshop is shown only as an example of a reproducible workflow. The procedure is fully detailed in Supplementary S1, Section 2.2, with annotated screenshots (Figures S1-A to S1-E). This approach matches other proportional 2D measurements commonly used in facial, orthodontic, and soft-tissue research.

3. AIS (Armenti Inversion Scale) Illustrated Guide

3.1 Purpose

AIS is a **visual, descriptive aid** to communicate severity of visible upper lip inversion during smiling. It is **not a validated diagnostic tool**, and **does not imply muscular etiology**.

3.2 Grading (Reference Only)

AIS grades are presented as illustrative visual references to support descriptive communication of inversion severity. The scale is not intended to function as a rule-based classification system, and no fixed visual criteria, thresholds, or mandatory features are defined for each grade. Grading reflects a global visual impression of inversion severity, which may involve central, lateral, or combined

vermilion regions. AIS grades should not be interpreted as ordinal thresholds for clinical action or outcome assessment

Images S1-F

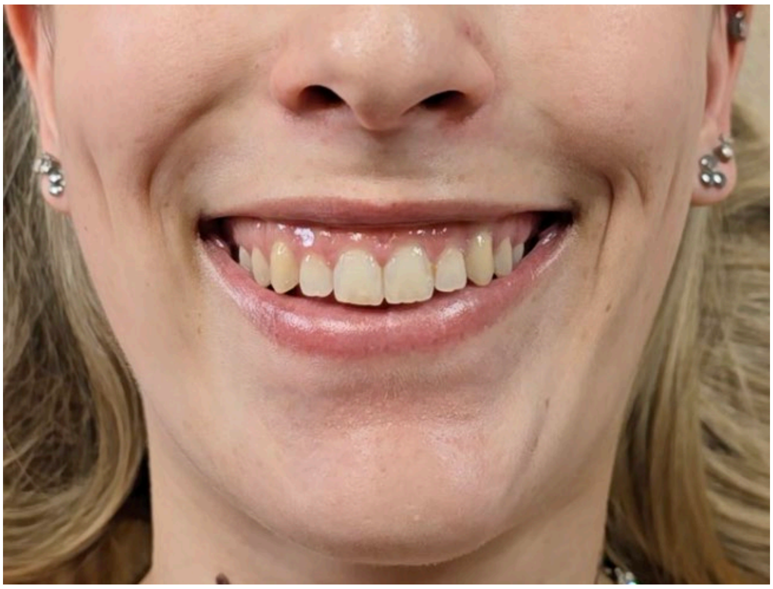

Caption: AIS-1 - Illustrative example of mild inversion during smiling, characterized by limited inward rolling and overall preservation of visible vermilion. This image is provided as a visual reference only and does not define mandatory features or diagnostic criteria.

Images S1-G

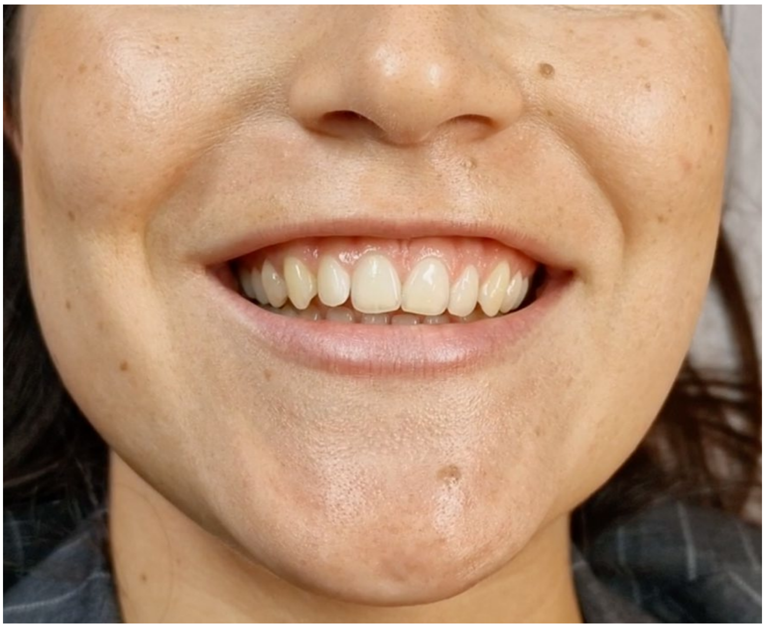

Caption: AIS-2 - Illustrative example of moderate inversion during smiling, showing evident reduction of visible vermilion with partial mucosal exposure. This image represents a typical visual impression and is not intended to establish fixed grading thresholds.

Images S1-H

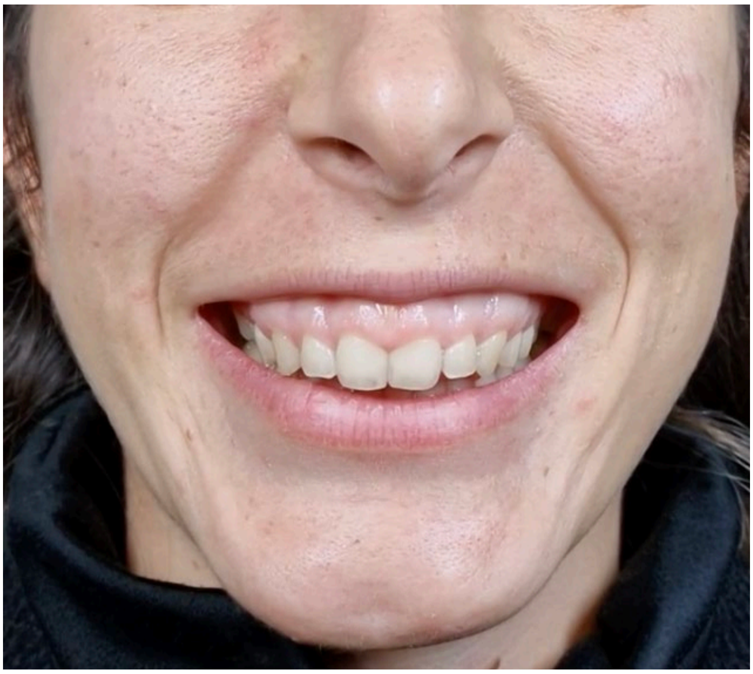

Caption: AIS-3 - Illustrative example of severe inversion during smiling, with pronounced inward rolling and extensive mucosal or gingival visibility. The image is illustrative only and does not imply obligatory visual hallmarks or diagnostic boundaries.

3.3 Recommended Use

- Reported **only for exploratory categorization**
- Used **without thresholds for treatment**
- Intended to support **comparative research**, NOT classification of patients

AIS must **not** be used to define:

- clinical diagnosis
- muscle selection certainty
- treatment indication or dosage recommendation

4. Common Observational Pitfalls (Illustrative)

The following notes highlight frequent sources of observational inconsistency identified during the present study and are not intended as prescriptive rules.

Avoid	Correct Approach
Measuring mucosa as vermilion	Stop at **visible red border**
Using non-vertical lines	Use **Shift** to fix vertical direction
Scaling images manually	**Never resize independently**
Treating AIS as diagnosis	AIS = **visual label only**
Interpreting ΔLv% causally	ΔLv% does **not prove muscular origin**

5. Summary Statement

This workflow provides standardized documentation and exploratory quantification of vermilion inversion during smiling. AIS and ΔLv% are descriptive tools without diagnostic validity. They serve to improve reproducibility of future observational and comparative studies, and do not justify therapeutic decisions or establish muscular mechanisms. The regionalized ΔLv% approach is specifically intended to reduce underestimation of lateral-dominant inversion patterns and to maintain anatomical coherence with marginal intraoral injection strategies.

File MOOH DeltaLV Exploratory.xlsx

This spreadsheet provides a pre-formatted template for exploratory quantification of upper lip vermilion reduction during smiling (ΔLv%). The tool is designed to support reproducible proportional measurements in observational research.

The file contains:

- pre-set input fields for visible vermilion length at rest and during smiling (pixel units, red vermilion only, mucosa excluded),
- an automated, locked ΔLv% formula,
- optional AIS observational fields (non-validated), and
- a permanent disclaimer indicating that ΔLv% and AIS are **exploratory and non-diagnostic**.

The formula used to compute ΔLv% is:

$$\Delta Lv\% = \frac{Lv_{rest} - Lv_{smile}}{Lv_{rest}} \times 100$$

Pixel units are acceptable because the measurement expresses relative change in vermilion visibility. The spreadsheet is provided to improve reproducibility of research reporting and does not imply diagnostic validity or treatment recommendation.
