# Supplementary material for: Marginal Orbicularis Oris Hyperactivity (MOOH): An Exploratory Case Series of Inversion-Dominant Upper Lip Dynamics Following Intraoral Botulinum Toxin Type A
Source: Toxins (Basel). 2026 Mar 17;18(3):146. doi: 10.3390/toxins18030146 (PMC13029986; doi:10.3390/toxins18030146)
Supplement: Supplementary file 1 [file toxins-18-00146-s001.zip › Supplementary Material S2.pdf]

Supplemetary Materials – S2

Supplementary Tables: Extended Descriptive Outcomes, Sensitivity Analyses, and Functional Safety Data

This appendix provides supplementary tables reporting extended descriptive outcomes, exploratory concordance analyses, sensitivity analyses, and detailed functional safety data derived from the present case series.

The tables included in this section are intended to support transparency, reproducibility, and methodological robustness by providing granular data that complement the main manuscript. These materials do not introduce additional endpoints, diagnostic criteria, or treatment recommendations, nor are they intended to guide clinical decision-making. All analyses reported in Supplementary Material S2 are exploratory and descriptive in nature and should be interpreted within the context of the study's non-comparative design and predefined methodological constraints.

Table S1. SS-PRO* (Aesthetic and Functional Patient-Reported Outcomes)

Domain (Item)	Score Range	Baseline (Mean ± SD)	Day 15 (Mean ± SD)
**Aesthetic satisfaction**	0–10	2.3 ± 1.1	7.8 ± 1.4
**Self-perceived inversion**	0–10	8.7 ± 1.2	3.2 ± 0.9
**Smile confidence**	0–10	-	-
**Lip competence during speech**	0–10	-	-
**Lip competence during suction**	0–10	-	-

**The SS-PRO is an exploratory, study-specific tool and has not undergone formal psychometric validation.*

Legend: Study-Specific Patient-Reported Outcome (SS-PRO) is a non-validated exploratory instrument designed to capture patient-perceived aesthetic and functional changes following intraoral marginal chemodenervation. The scale includes five items scored on a 0–10 range. Numerical values (mean ± SD) are reported only for items that were explicitly quantified and analyzed (aesthetic satisfaction and self-perceived inversion). For items related to smile confidence and functional competence (speech and suction), no clinically relevant variation was detected between baseline and Day 15; therefore, these items are reported qualitatively as stable.

Table S2. Combined Aesthetic–Functional Treatment Outcome Classification

Outcome Category	Definition	Patients (n, %)
**Optimal Outcome**	AIS 0 + no functional impairment	26 (27.7%)
**Effective Outcome**	AIS 1 + no functional impairment	48 (51.0%)
**Compensated Outcome**	AIS improvement ≥1 grade + transient functional impairment (fully resolved)	17 (18.1%)
**Undesirable Outcome**	AIS improvement <1 grade and/or persistent functional impairment	3 (3.2%)

Legend: Composite endpoint combining observed AIS improvement and functional integrity. This measure provides a more holistic assessment of overall outcome beyond isolated cosmetic change.

Table S3. Internal Consistency Between AIS and ΔLv% (Exploratory Concordance Analysis)

Analysis Domain	Variables Compared	n	Spearman ρ	95% CI	p-value	Interpretation (descriptive)
**Baseline concordance**	AIS grade (0–3) vs ΔLv% at baseline	94	0.74	0.62–0.82	<0.001	Higher AIS grades were associated with higher ΔLv% (greater vermilion loss), supporting monotonic alignment between qualitative and quantitative descriptors
**Early-response concordance**	ΔAIS (Baseline→Day 15) vs ΔΔLv% (Baseline→Day 15)	94	0.68	0.54–0.78	<0.001	Larger AIS grade improvements were associated with larger proportional reductions in ΔLv%, supporting directional coherence of change metrics
**Cross-sectional at Day 15**	AIS at Day 15 vs ΔLv% at Day 15	94	0.71	0.58–0.80	<0.001	Residual inversion grading remained aligned with residual quantitative vermilion loss at short-term follow-up

Legend: Exploratory concordance between AIS and ΔLv% using Spearman rank correlations. These analyses describe internal consistency between qualitative grading and quantitative vermilion loss measures and do not constitute diagnostic validation. ΔLv% represents the composite regional proportional reduction of visible vermilion from rest to maximal smile, calculated as the arithmetic mean of three regional values (central and bilateral lateral segments) as defined in the Methods.

Table S4. Sensitivity Analysis of the Operational Vertical Excursion Cutoff

Table S4A. Distribution of cases under alternative vertical excursion thresholds

Operational threshold definition	Cases retained under operational threshold (n, %)	Notes
**≤ 1.5 mm**	78 (83.0%)	More restrictive threshold; retains the most "pure" minimal-elevation subgroup
**≤ 2.0 mm (main analysis)**	94 (100%)	Pre-specified operational cutoff used in the manuscript
**≤ 2.5 mm**	94 (100%)	Permissive threshold; identical to main analysis in this cohort

Legend: Distribution of patients according to alternative operational thresholds for vertical upper lip excursion used for descriptive sensitivity analyses. More restrictive (≤1.5 mm) and more permissive (≤2.5 mm) thresholds were explored to assess the robustness of inversion-dominant classification. Thresholds reflect operational definitions used to retain or restrict cases within the analytical set and do not represent measured prevalence of vertical excursion or diagnostic boundaries.

Table S4B. Robustness of key outcomes across thresholds

Threshold group	n	AIS 0 at Day 15 n (%)	Residual AIS 2 at Day 15 n (%)	Mean ΔLv% at Day 15 ±SD	Descriptive summary
**≤ 1.5 mm**	78	25 (32.1%)	9 (11.5%)	39 ± 10	Comparable short-term modulation patterns to the main cohort; no material shift in outcome direction
**≤ 2.0 mm (main)**	94	29 (30.9%)	11 (11.7%)	40 ± 10	Primary cohort results as reported
**≤ 2.5 mm**	94	29 (30.9%)	11 (11.7%)	40 ± 10	Identical to main analysis

Legend: Short-term qualitative (AIS grade distribution) and quantitative (ΔLv%) outcomes at Day 15 across alternative operational thresholds for vertical excursion. Comparable inversion modulation patterns across thresholds indicate that the observed findings were not driven by the specific cutoff selected. Results are presented descriptively and do not imply validation of vertical excursion thresholds or treatment efficacy.

Table S5. Functional Safety and Adverse Events

Functional Domain	Impairment Evaluated	Patients affected (n)	Severity	Resolution Time
**Speech**	Distortion of bilabials (P/B/M)	8	Grade 1–2	Approx. 15 days
**Oral competence**	Drooling	4	Grade 1–2	Approx. 15 days
**Feeding**	Difficulty suctioning/retaining fluids	3	Grade 1–2	Approx. 16 days
**Kissing**	Reported alteration	5	Grade 1	Approx. 16 days
**Aesthetic - functional**	Asymmetry, excessive weakness	3	Grade 1–3	Approx. 7 days

Legend: Functional evaluation adapted from established neuromuscular safety criteria for perioral BoNT-A. All events were mild-to-moderate, transient, and self-resolving without medical intervention.

Table S6. Functional Safety Outcomes Stratified by Vermilion Recovery

ΔLv% Recovery Category (Day 15)	Patients (n)*	Speech Alteration (patients, n)	Suction Difficulty (patients, n)	Drooling (patients, n)	Kissing Alteration (patients, n)
**≥ 80% ΔLv% Recovery**	29	1	0	1	1
**60–79% Recovery**	54	4	1	2	2
**< 60% Recovery**	11	3	2	1	2

* Recovery categories reflect each patient's proportional reduction in ΔLv% from baseline to Day 15, stratified into ≥80%, 60–79%, and <60% recovery bands.

Legend: Functional adverse events stratified by degree of vermilion recovery. Event frequencies are reported as number of patients affected within each recovery category. No causal or predictive inference is implied.
